# Kanglaite inhibits EMT caused by TNF-α via NF-κΒ inhibition in colorectal cancer cells

**DOI:** 10.18632/oncotarget.23645

**Published:** 2017-12-22

**Authors:** Guiling Shi, Xiaoli Zheng, Shiwu Zhang, Xiaojing Wu, Fei Yu, Yijia Wang, Fei Xing

**Affiliations:** ^1^ Tianjin Union Medical Center, Tianjin 300121, China; ^2^ The Key Laboratory of Weak Light Nonlinear Photonics, Ministry of Education, Teda Applied Physics School and School of Physics, Nankai University, Tianjin 300071, China; ^3^ School of Pharmacy, Tianjin Medical University, Tianjin 300121, China

**Keywords:** TNF-α, EMT, kanglaite, NF-κΒ, colorectal cancer cell lines

## Abstract

Tumor necrosis factor-alpha is a critical pro-inflammatory cytokine produced by macrophages and was once considered an anti-tumor agent. However, a low dose of tumor necrosis factor-alpha can cause epithelial mesenchymal transition, angiogenesis and metastasis. NF-κΒ contributes to epithelial mesenchymal transition induced by tumor necrosis factor-alpha. Kanglaite, an extract from the Coix lacryma-jobi (adlay) seed, is an NF-κΒ inhibitor. The aim of this study was to investigate whether Kanglaite could inhibit epithelial mesenchymal transition caused by tumor necrosis factor-alpha using four colorectal cancer cell lines, HCT106, HCT116, LoVo and CT26. Our results showed that tumor necrosis factor-alpha -mediated activation of NF-κΒ, caused changes in epithelial mesenchymal transition -related protein expression, and increased migration and invasion in all four cell lines. However, these effects were inhibited by Kanglaite when used in combination with tumor necrosis factor-alpha. In a subcutaneous tumor model of CT26, tumor necrosis factor-alpha enhanced the tumorigenic ability of the cells, and again this was inhibited by Kanglaite. However, treatment with Kanglaite alone caused almost no inhibition of epithelial mesenchymal transition -mediated tumor growth, when cells were pretreated with tumor necrosis factor-alpha prior to injection. These results suggest that Kanglaite inhibits tumor necrosis factor-alpha -mediated epithelial mesenchymal transition in colorectal cancer cell lines via inhibition of NF-κΒ.

## INTRODUCTION

Tumor necrosis factor-alpha (TNF-α) is a critical pro-inflammatory cytokine that has been shown to play an important role in many physiological and pathological processes, including immunity, cachexia, inflammatory reaction, and tumor progression [1]. As a multifunctional cytokine, TNF-α has a complex role in the treatment of cancer. High doses of TNF-α can inhibit tumor angiogenesis and induce apoptosis, but long-term treatment with low doses causes proliferation, invasion, angiogenesis and metastasis of tumor cells [2]. It is also worth mentioning that TNF-α may be related to cancer development and metastasis in certain tumor microenvironments [3].

Furthermore, as an endogenous tumor-promoting factor, TNF-α can also cause epithelial mesenchymal transition (EMT) in several types of cancer cells [4]. Overproduction of TNF-α alters the adhesive ability of tumor cells and enhances their metastatic potential. It has been confirmed that TNF-α expression levels have a positive correlation with Dukes’ stages, and that TNF-α mRNA transcripts are higher in colorectal cancer (CRC) cells than normal epithelial colon cells [5]. This correlation between TNF-α and tumor grade is also seen in severe ovarian tumors [6].

TNF-α activates signaling pathways such as NF-κΒ, which correlates with TNF-α-mediated tumor cell invasion and migration [7]. Upon TNF-α stimulation, activated IKKβ phosphorylates IκΒα, an NF-κΒ inhibitor, then induces its degradation and triggers the liberation of NF-κΒ. As a result, the NF-κΒ heterodimer translocates to the nucleus and induces the expression of genes related to cell proliferation, cell survival, invasion, EMT, immune response and angiogenesis. Because NF-κΒ activation induces EMT [8], NF-κΒ is a potential target to prevent tumor cell invasion and migration induced by some agents.

NF-κΒ is essential for TNF-α-induced EMT [9]. Research has shown that the invasion and migration induced by TNF-α can be inhibited by drugs that inhibit NF-κΒ activation. For example, vitamin D3 upregulates protein 1, which inhibits hepatocarcinogenesis by suppressing TNF-α induced NF-κΒ activation [10]. Crebanine can also suppress invasion of human lung adenocarcinoma cells by blocking NF-κΒ-regulated gene products [11].

It has been reported that Kanglaite (KLT) inhibits NF-κΒ signaling [12, 13]. KLT, a standardized, pharmaceutical-grade emulsion of Coix seed oil, has been approved by the Chinese Ministry of Public Health for several years based on its clinical evidence for broad-spectrum anti-tumour activity [14–17]. KLT has been used in to treat over millions patients in China for treatment of various common types of cancer [18]. The main active ingredient of KLT is a triglyceride containing four types of fatty acids. Research has shown that KLT principally blocks cell cycle at G2/M phase to reduce cellular mitotic division and inhibit proliferation of tumour cells. KLT can also activate pro-apoptotic factors and lead cells to apoptosis [6]. It is known that KLT [13] or Coix seed extract [12] can inhibit NF-κΒ, but whether KLT inhibits EMT is still unclear. Therefore, we hypothesized that KLT may suppress the invasion and migration induced by TNF-α in cancer cells. Four CRC cell lines were used to investigate the potential inhibitory effect of KLT on TNF-α induced cell invasion and migration in colorectal cancer.

## RESULTS

### Determination of suitable TNF-α and KLT concentrations

The impact of TNF-α and KLT on cell viability was assessed using an MTT assay. Supplementary Figure 1 shows that TNF-α at concentrations above 20 ng/ml had a considerable inhibitory effect on cell viability. However, lower concentrations of TNF-α (<5 ng/ml for CT26, and <10 ng/ml for the other three cell lines) did not exhibit obvious cytotoxic effects, even after a relatively long treatment time (48 h). Within the scope of concentrations used in this assay, KLT only had a low inhibitory effect. When TNF-α and KLT were used together, the cytotoxic effect of TNF-α was slightly enhanced by the presence of KLT. Therefore, for all subsequent experiments, a concentration of 10 ng/ml TNF-α and 2 μl/ml KLT was used for the HCT106, HCT116 and LoVo cell lines, and 5 ng/ml TNF-α and 1 μl/ml KLT was used for the CT26 cells.

### KLT inhibits TNF-α-induced NF-κΒ activation

NF-κΒ is a key transcriptional regulator of extracellular proteases and pro-inflammatory cytokines that promote survival, invasion and adhesion of cancer cells [18, 19]. NF-κΒ activation also contributes to EMT, which is important for cancer dissemination [20]. NF-κΒ exists as a homodimer or as a heterodimer composed of two subunits, p50 and p65. The p50-p65 dimer maintains normal physiological function *in vivo*. Therefore, we used an anti-p65 antibody to identify NF-κΒ activation. Normally, NF-κΒ is sequestered by members of the IκB family to form an inactivated compound in the cytoplasm. Stimulation of cells with TNF-α initiates a cascade of signaling events leading to NF-κΒ activation. After TNF-α binds to the TNF receptor, IκB kinase (IKK) is activated, which causes IκB degradation. When NF-κΒ dissociates from IκΒ, NF-κΒ is activated and transferred to the nucleus. IKK and IκΒ are both are cytoplasmic proteins; therefore, they can only be detected in cytosolic extractions [21].

**Figure 1:**
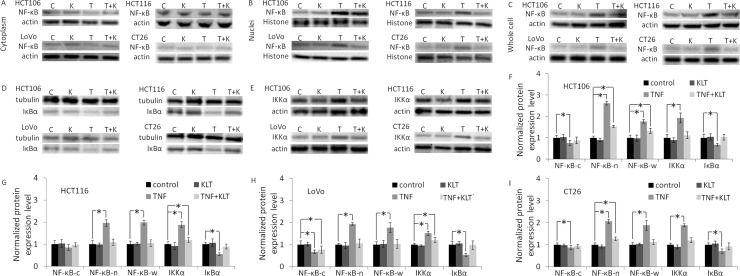
Western blot analysis of NF-κΒ p65, IKKα and IκBα expression NF-κΒ expression was measured in the cytoplasm, nuclei and whole cell lysate in order to determine whether NF-κΒ is translocated to the nucleus induced by TNF-α. Because IKK and IκB are both cytoplasmic proteins, and also interact with NF-κΒ in the cytoplasm, these proteins could only be detected in the cytoplasmic samples. In blot images, C represents control, K represents KLT only, T represents TNF-α only and T+K represents TNF-α plus KLT. Experiments were performed in triplicate. (**A**) NF-κΒ p65 expression in the cytoplasm. (**B**) NF-κΒ p65 expression in the nuclei. (**C**) NF-κΒ p65 expression in whole cells. (**D**) IκBα expression in the cytoplasm. (**E**) IKKα expression in the cytoplasm. (F–I) Bar diagrams of densitometric analysis of NF-κΒ, IKKα and IκΒα expression in the four CRC cell lines. NF-κΒ-c represents NF-κΒ expression in cytoplasm, NF-κΒ-n represents NF-κΒ expression in nuclei, and NF-κΒ-w represents NF-κΒ expression in the whole cell. ^*^P < 0.05 indicates a significant difference compared to the control group. Full length blots of A–E are shown in Supplementary Figure 5.

Figure 1 and Supplementary Figure 2 show that TNF-α activated NF-κΒ, decreased IκB expression and upregulated IKK expression in all four CRC cell lines. These results are similar to published research [9, 11]. KLT inhibited the effect of TNF-α, but when KLT was used alone it had no significant effect on NF-κΒ, IKK or IκB expression.

### TNF-α increases EMT-related protein expression, which is inhibited by KLT

**Figure 2:**
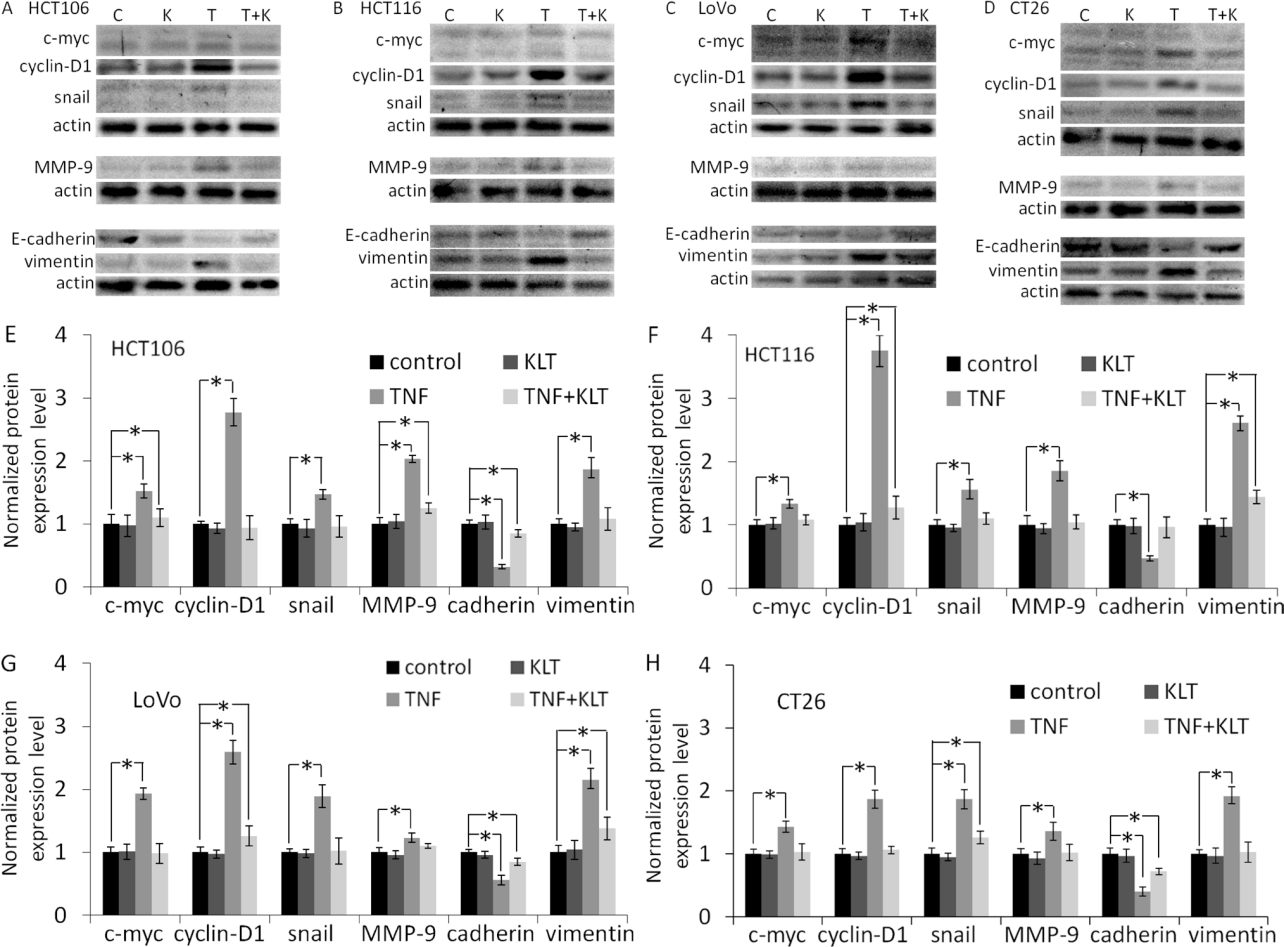
Western blot analysis of c-myc, cyclin-D1, snail, MMP-9, E-cadherin and vimentin expression Experiments were performed in triplicate. (**A**–**D**) Protein expression in the four CRC cell lines after treatment with different reagents, as indicated. C represents control, K represents KLT only, T represents TNF-α only, and T+K represents TNF-α plus KLT. (**E**–**H**) Bar diagrams of densitometric analysis of data in panels A–D. ^*^P < 0.05 indicates a significant difference compared to the control group. Full length blots of A–D are shown in Supplementary Figure 6.

NF-κΒ activation is a reciprocal response to various anti-tumor reagents [22], and TNF-α is no exception [23]. NF-κΒ upregulates proteins that promote tumor growth and proliferation, such as cyclin-D1, c-myc [23], MMP-9 [24]. TNF-α also potentiates EMT, for example by upregulating vimentin, stabilizing snail, or downregulating E-cadherin [25]. Our results (Figure 2 and Supplementary Figure 3) showed similar effects in that cyclin-D1, c-myc, MMP-9, vimentin and snail expression were all increased, while E-cadherin expression was decreased in the four CRC cell lines after treatment with TNF-α. KLT treatment alone had no significant effect on the expression of these proteins. But when cells were treated simultaneously with TNF-α and KLT, KLT inhibited these changes in protein expression induced by TNF-α. In addition, after 48 h treatment with TNF-α (Supplementary Figure 4), the HCT106, HCT116 and LoVo cells exhibited a change in morphology, from an epithelial morphology to a mesenchymal spindle-like shape, with fusiform features. The CT26 cells exhibited no obvious morphological changes, which may due to their already fibrous morphology. KLT was able to reverse these morphological changes when combined with TNF-α.

### KLT inhibits the migration and invasion of CRC cells promoted by TNF-α

**Figure 3:**
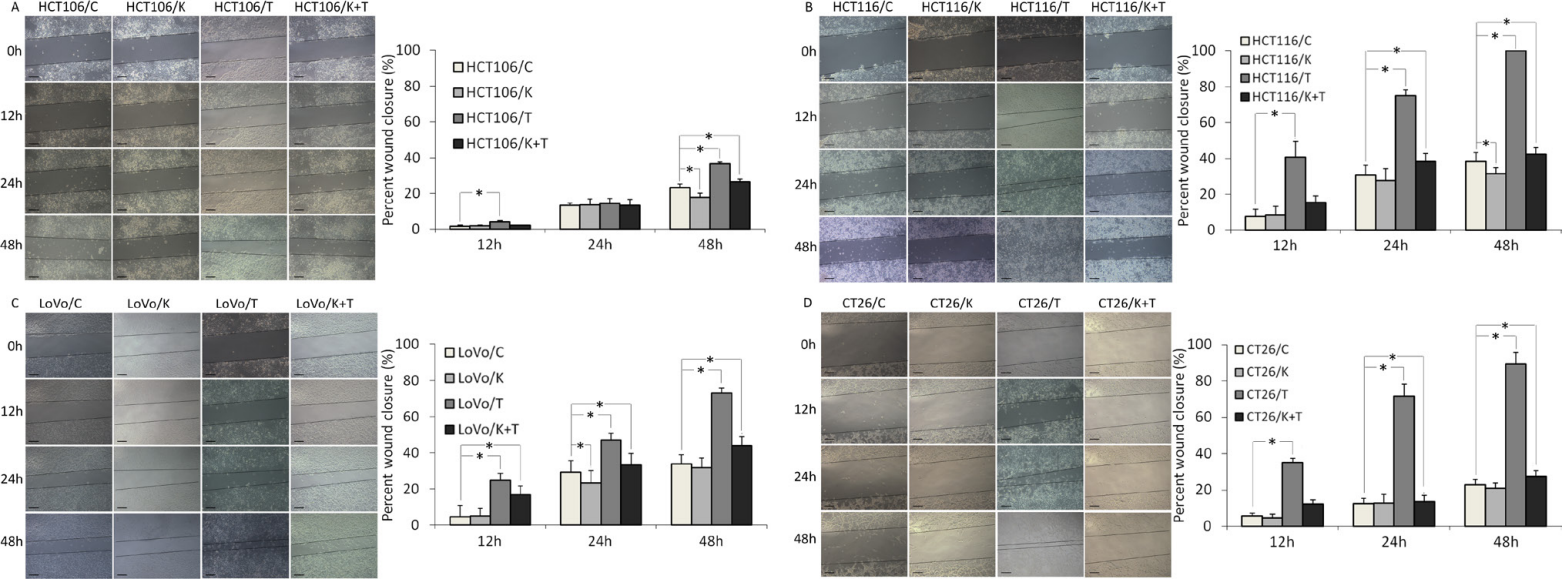
Migration of the four CRC cell lines after KLT and TNF-α treatment was measured using a wound healing scratch assay (original magnification ×100) C represents control, K represents KLT only, T represents TNF-α only, and T+K represents TNF-α plus KLT. The scale bars represent 100 μm. Experiments were performed in triplicate. ^*^P < 0.05 indicates a significant difference compared to the control group. Furthermore, for all cell lines, the percent wound closure in the K+T group was significantly lower than for the T group (*P* < 0.05).

**Figure 4:**
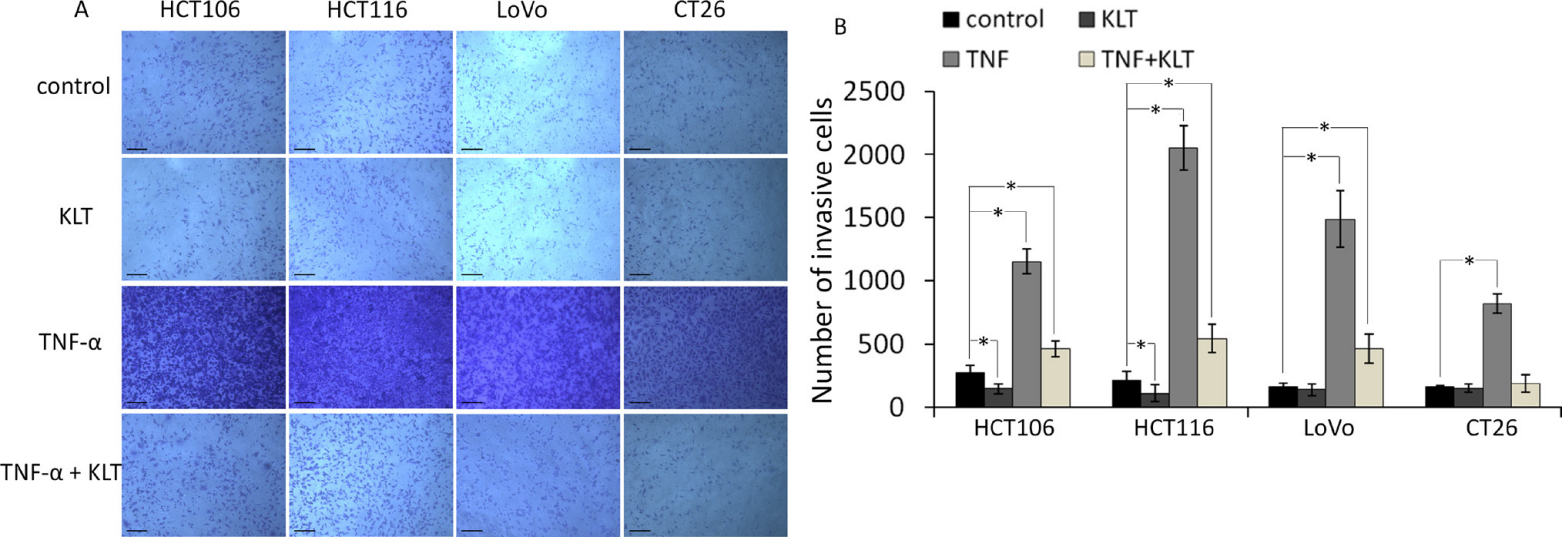
Invasion of the four CRC cell lines after KLT and TNF-α treatment was measured using a transwell assay (original magnification ×100) The scale bars represent 100 μm. Experiments were performed in triplicate. ^*^P < 0.05 indicates a significant difference compared to the control group. For all cells lines, invasion cell numbers were significantly lower in the TNF+KLT group than in the TNF group (*P* < 0.05).

It has been reported that TNF-α can enhance the migration and invasion abilities of cancer cells [25]. To investigate whether KLT exerts a regulatory effect on cell migration and invasion induced by TNF-α, wound healing scratch and transwell invasion assays were performed. As shown in Figure 3, the four CRC cell lines exhibited significantly enhanced migration at 48 h after TNF-α treatment. When cells were treated simultaneously with TNF-α and KLT, this enhancement effect was inhibited. As shown in Figure 4, the transwell invasion assay also revealed that TNF-α promoted the invasive ability of these cells, while KLT inhibited this effect. In addition, treatment with KLT alone had a slight inhibitory effect on cell migration and invasion.

### TNF-α enhances the tumorigenic ability of CT26 cells, while KLT inhibits it *in vivo*

**Figure 5:**
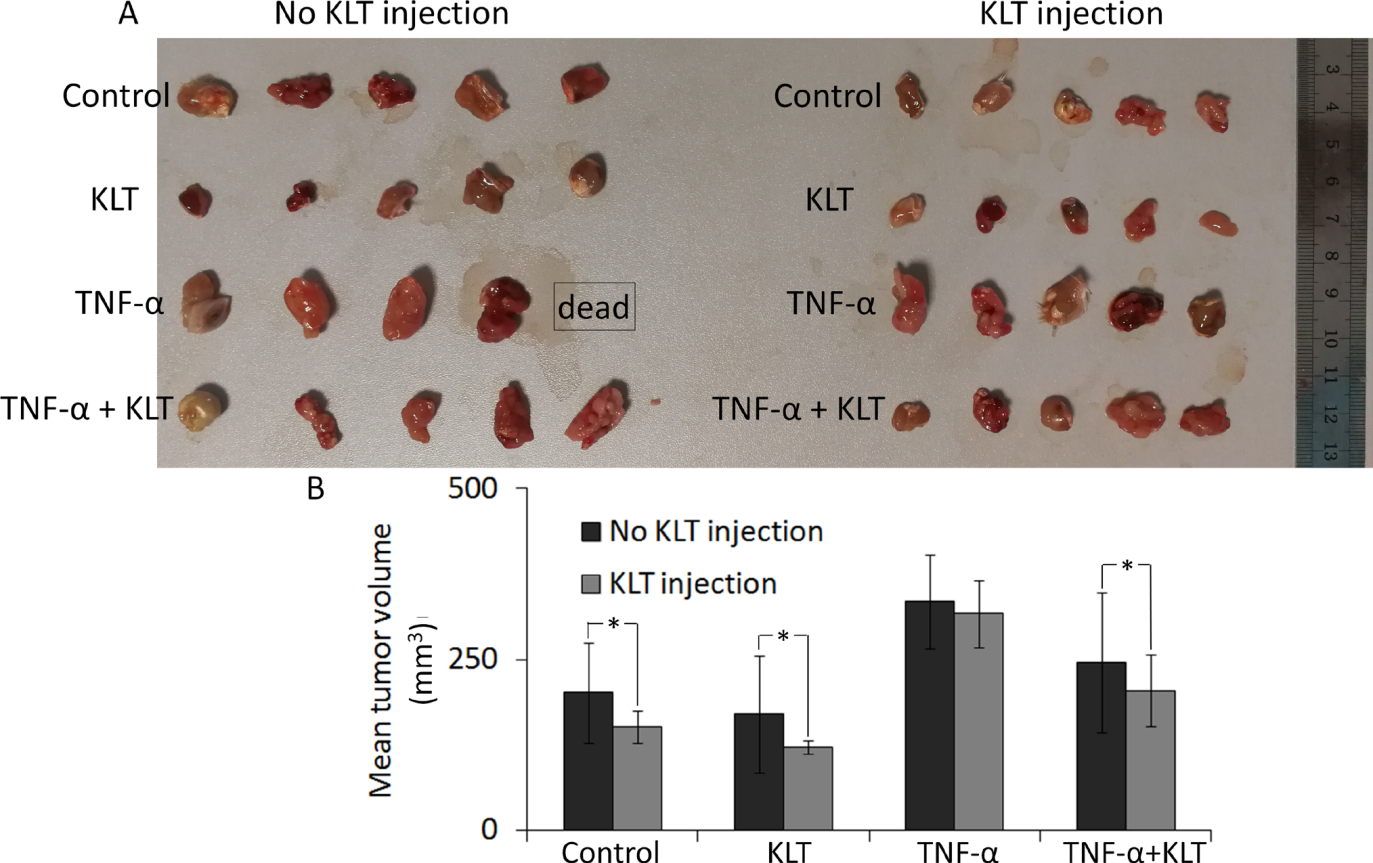
Inhibition of tumor growth by KLT (**A**) Four different pretreatment strategies of CT26 cells used for subcutaneous inoculation. Each group contained five mice. Tumors were allowed to develop for 15 days, but one mouse from the TNF-α pretreated & no KLT injection group was found dead at 14 days. (**B**) Columns represent mean volume from 5 tumors, except for the TNF-α pretreated & no KLT injection data, which came from 4 tumors. Average tumor volumes in two adjacent columns were compared using the Student's *t*-test. ^*^P < 0.05 represents a significant difference.

Compared with tumors acquired from tumor-bearing mice, it was found that KLT treatment inhibited the tumorigenic ability of CT26 cells (Figure 5). Briefly, in mice that did not receive a KLT injection, the TNF-α pretreated group developed the largest tumors, which may due to EMT caused by TNF-α. Tumor volume in the TNF-α + KLT group was reduced, suggesting that KLT offsets part of TNF-α effect. A single KLT pretreatment also exhibited some inhibition of tumorigenic ability. On the other hand, tumor growth was slightly reduced in the KLT injected mice, but KLT had only a mild inhibitory effect on TNF-α-induced EMT.

## DISCUSSION

Many epithelial tumors undergo EMT, which facilitates their invasion. Longterm treatment with anti-tumor drugs, such as paclitaxel [26] and cisplatin [27], causes tumor resistance and EMT, but some agents can reverse the EMT induced by anti-tumor drugs [26, 27]. It has been shown that NF-κΒ activation is one response of tumor cells to some drugs, and that it contributes to EMT [8, 28]. TNF-α induces EMT via many signal pathways, including NF-κΒ [9], and our results showed that KLT, an NF-κΒ inhibitor, was able to inhibit the EMT induced by TNF-α.

As an anti-tumor agent, TNF-α induces cancer cell death, and KLT also has anti-tumor effects. Therefore, we used an MTT assay to determine suitable concentrations for TNF-α and KLT treatment to obtain an inhibition rate of about 10% after 48 h. Low levels of cytotoxicity using these combined drugs facilitated the analysis EMT in four CRC cell lines in subsequent experiments.

EMT can be triggered by signals from the tumor microenvironment. TNF-α is produced by macrophages and activates inflammatory processes during tumor promotion [29]. NF-κΒ is downstream of TNF-α and other inflammatory cytokines produced in the tumor microenvironment, and is a major mediator of the tumor-promoting activity of inflammatory cytokines [30]. NF-κΒ signaling is related to TNF-α-induced EMT [7, 9]. Therefore, if an agent inhibits NF-κΒ signaling, it may also inhibit the invasion and migration that was induced by TNF-α. Previous research showed that KLT inhibits NF-κΒ activation [12, 13], and our results also showed that TNF-α activated NF-κΒ in four CRC cell lines, whereas KLT inhibited NF-κΒ. We found that KLT upregulated IKKα at TNF-α treated cells, which suggested that KLT may inhibit NF-κΒ activation on blocking upstream proteins, for example, PI3K/Akt or TNF receptor, but the mechanism of how KLT inhibits on these proteins remains to be further investigated. Several EMT-related proteins were measured and were found to be upregulated by TNF-α, whereas KLT was shown to partially offset the effects of TNF-α. The wound healing scratch and cell invasion assays confirmed that TNF-α increased the migration and invasion ability of the cells, and that this was offset by KLT. Treatment with KLT only had almost no effect on migration, but did have some inhibitory effect on the invasion of HCT106 and HCT116 cells, but not LoVo and CT26 cells. These variable results could due to the low concentration of KLT, as different cells do not have the same sensitivity to KLT.

Unlike the other three CRC cell lines that have an epithelioid morphology, CT26 cells exhibit a fibrous morphology, but nonetheless are commonly used to research EMT *in vitro* and *in vivo* [31]. Our MTT assay showed that TNF-α is more cytotoxic in CT26 than in other cells, but that CT26 also exhibited variable changes in EMT-related protein expression at lower concentrations of TNF-α treatment. Our subcutaneous tumor model showed that CT26 cells with a more EMT-like phenotype had more tumorigenic ability, and that the tumor volume decreased significantly after an intraperitoneal injection of KLT. The wound healing scratch and cell invasion assays showed that KLT had almost no effect on the migration and invasion abilities of CT26 cells *in vitro*, which could be due to the low concentration of KLT that was used to treat the cells. The KLT concentration used in our *in vitro* experiments was relatively low [32] in order to avoid significant cytotoxicity, because the NF-κΒ inhibitor may sensitize cells to TNF-α [33]. Intraperitoneal injections of a higher concentration of KLT were used to inhibit tumor growth *in vivo*, according to a previous report [13] and our preliminary experiments. At this concentration, KLT exhibited an inhibitory effect on tumor growth *in vivo,* which suggests that KLT has a mild effect on tumors with EMT *in vivo*. Thus, KLT may have a preventive effect on EMT development but does not inhibit EMT tumor formation.

## CONCLUSIONS

The present work investigated the inhibitory effect of KLT on TNF-α-induced EMT. Four CRC cell lines, HCT106, HCT116, LoVo and CT26, were used in both *in vitro* and *in vivo* experiments. Our results showed that TNF-α activated NF-κΒ and induced EMT in all four of the cell lines. When cells were treated with a combination of TNF-α and KLT, the NF-κΒ activation was inhibited, EMT-related protein expression was decreased, and the migration and invasion effects caused by TNF-α were accentuated. However, when the cells were treated with KLT alone, these effects were mild. A Balb/c mouse model was used to determine the tumorigenic ability of CT26 cells in combination with different agents and we drew the same conclusions. Furthermore, KLT had an inhibitory effect on tumor growth *in vivo,* but also had a slight inhibitory effect on tumors derived from the TNF-α group, suggesting that KLT is not effective against EMT tumors. In summary, our data suggest that KLT prevents EMT caused by TNF-α via inhibition of NF-κΒ signaling.

## MATERIALS AND METHODS

### Reagents and antibodies

All cell culture media, antibiotics, and trypsin were purchased from Gibco (Grand Island, NY, USA), and fetal bovine serum (FBS) was purchased from HyClone (Logan, UT, USA). Mouse anti-E-cadherin antibody, mouse anti-NF-κΒ p65 antibody, mouse anti-cyclin D1 antibody, mouse anti-c-myc antibody, and mouse anti-snail antibody were purchased from Santa Cruz Biotechnology (CA, USA). Rabbit anti-matrix metalloproteinase-9 (MMP-9) antibody, rabbit anti-IKKα antibody, rabbit anti-IκΒα antibody, rabbit anti-vimentin antibody, rabbit anti-Histone H3 antibody, mouse anti-β-tubulin antibody, goat anti-rabbit IgG-peroxidase, goat anti-mouse IgG-peroxidase, goat anti-mouse IgG FITC, goat anti-rabbit IgG FITC, goat anti-mouse IgG TRITC, DAPI and methyl thiazolyl tetrazolium (MTT), were purchased from Sigma-Aldrich (St. Louis, MO, USA). Immobilon membranes were purchased from Merck Millipore (Bedford, MA, USA). ECL Plus substrate, bicinchoninic acid reagents, and RIPA lysis buffer were purchased from CWBio (Beijing, China). TNF-α was purchased from Sino biological Inc. (Beijing, China). KLT injection was purchased from Zhejiang Kanglaite Pharmaceutical Co., Ltd, (Zhejiang, China). Nuclear and cytoplasmic protein extraction kit, crystal violet were purchased from Beyotime (Shanghai, China).

### Cell lines

HCT106, HCT116, LoVo, and CT26 cell lines were purchased from the Shanghai Institutes for Biological Sciences, Chinese Academy of Sciences. HCT106, HCT116, and LoVo cell lines are human CRC cell lines. CT26 is a murine colon adenocarcinoma cell line derived from N-nitroso-N-methylurethane treated Balb/c mice. All cells were cultured in RPMI 1640 medium supplemented with 10% FBS, 100 μg/ml streptomycin and 100 U/ml penicillin.

### TNF-α and KLT treatment

The four cell lines were treated with different concentrations of TNF-α and KLT in order to identify a suitable concentration that caused no significant cytotoxicity. Four treatment groups were used: (1) Cells without drug treatment served as the control group; (2) Cells treated with KLT alone for 48 h; (3) Cells treated with TNF-α alone for 48 h; (4) Cells treated with a mixture of TNF-α and KLT for 48 h. Cell viability was measured using an MTT assay. The rate of cell growth inhibition in each well was calculated by defining the absorption of the control group as 100%. Measurements were performed in triplicate. Cells from all four groups were used for western blot, immunocytochemistry, the wound healing scratch assay and the cell invasion assay.

### Western blot analysis

Cells from all four experimental groups were trypsinized and used to generate protein samples to detect NF-κΒ, IKKα, IκΒα, E-cadherin, vimentin, MMP-9, snail, cyclin D1 and c-myc expression.

To detect NF-κΒ, IκΒα and IKKα expression, nuclear and cytosolic proteins were separated from the harvested cells using a nuclear and cytoplasmic protein extraction kit (Beyotime, Shanghai, China) according to manufacturer's instructions. Briefly, 200 μl cytosolic protein extraction agent A with 1 mM phenylmethyl sulfonylfluoride (PMSF) was added to 20 μl harvested cells at 4°C. The mixture was incubated on ice for 15 min then 10 μl cytosolic protein extraction agent B was added. Tubes were vortexed vigorously for 5 s then incubated on ice for 1 min. After 5 s vigorous vortexing, tubes were centrifuged at 15,000 × *g* for 5 min at 4°C. The supernatant, containing the cytoplasmic fraction, was transferred to a fresh tube. The insoluble pellet, containing the nuclear fraction, was resuspended in nuclear protein extraction agent (50 μl) with 1 mM PMSF, vortexed vigorously for 20 s and incubated on ice for 30 min. During this incubation, tubes were vortexed for 15 s every 5 min, for six times in total. Tubes were then centrifuged at 15,000 × *g* for 10 min at 4°C. The supernatant, containing the nuclear fraction, was transferred to a fresh tube.

To detect other proteins (E-cadherin, vimentin, MMP-9, snail, cyclin D1 and c-myc), 1 × 10^6^ cells were lysed with 500 ml RIPA lysis buffer containing a protein inhibitor for 20 minutes on ice. Cell lysates were centrifuged and supernatants, containing proteins, were transferred to fresh tubes.

Protein samples were quantitated using bicinchoninic acid reagents, resuspended in SDS-loading buffer and boiled. Protein (10 μg) was run on an SDS-PAGE gel and transferred to an Immobilon membrane using a semi-dry blotting method. The membrane was probed successively with appropriate primary and secondary antibodies using standard techniques. Finally, signals were visualized by ECL and membranes were exposed to film. Each assay was performed in triplicate. Expression levels were quantified using Quantity One software (Bio-Rad Laboratories, Inc., Hercules, CA, USA) and normalized against β-actin, β-tubulin or Histone H3, respectively.

### Immunocytochemistry analysis

Immunofluorescence staining was used to detect NF-κΒ, E-cadherin and vimentin expression. Briefly, cells were washed gently with PBS to remove culture medium, fixed with 10% formalin at room temperature for 15 minutes, treated with 0.5% Triton X-100 for 5 min at 4°C, then blocked overnight with 5% normal goat serum 4°C. Slides were washed with PBS and incubated with primary antibody for 1 h at 37°C. For NF-κΒ detection, slides were incubated with a mouse anti-NF-κΒ antibody. For E-cadherin and vimentin, slides were incubated simultaneously with mouse anti-E-cadherin and rabbit anti-vimentin antibodies. After washing with PBS, slides were incubated with a FITC- or/and TRITC-conjugated secondary antibody and DAPI for 1 h at 37°C. Finally, slides were washed with PBS and sealed. Coverslips were photographed immediately using an Olympus fluorescence microscope (Olympus, Japan).

### Wound healing scratch assay

Cells were cultured in 6-well plates until they formed a monolayer, then serum-starved overnight. An artificial scratch wound was created using a 10 μl pipette tip. The plate was washed with PBS to remove cell debris, then TNF-α and/or KLT was added to the cultures. Cells were maintained in serum-free culture medium at 37°C, in 5% CO_2_. Images of migration were captured at 0, 12, 24 and 48 h after scratching. Experiments were repeated independently in triplicate. The following equation was used to calculate percent wound closure: percent wound closure (%) = [1-(Lt/L0)] × 100%, where Lt represents width of scratch at time t and L0 represents initial width of scratch.

### Cell invasion assay

Cell invasion assays were performed using 24-well Matrigel-coated chambers (6.5 mm in diameter, 8 μm pore-size, 100 μg/cm^2^ Matrigel, Corning) according to the manufacturer's instructions. Briefly, cells were allowed to grow to subconfluency then serum-starved for 24 h. Precoated chambers were rehydrated, then cells were trypsinized and placed onto the upper transwell chamber (2 × 10^5^ cells per well) with 500 μl serum-free medium, with or without KLT/TNF-α. Medium (750 μl) containing 10% FBS was placed into the lower chamber. All conditions were repeated in triplicate. After 24 h incubation at 37°C in 5% CO_2_, cells that had not migrated were removed using a cotton swab, and cells that had migrated were fixed with 4% paraformaldehyde and stained with 0.1% crystal violet for 30 min. Images of three different fields were captured from each membrane.

### Subcutaneous tumor model

A subcutaneous tumor model was used to evaluate the tumorigenic ability of the CT26 cell line after treatment with different combinations of agents. The inhibitory effect of KLT on these tumors *in vivo* was also determined after intraperitoneal injection of KLT. Briefly, CT26 cells were treated with TNF-α and/or KLT for 48 h, at a concentration determined as described in section 3.1. Each group comprised 5 mice. Treated cells were trypsinized and approximately 1 × 10^6^ cells were injected subcutaneously into the flank of the mice (Six-week-old, Balb/c, females). An intraperitoneal injection of 6.25 μl/g KLT [13] was administered every day. Mice were sacrificed 15 days after cell implantation. Tumors were removed and size was measured using calipers. Tumor volume = L × S^2^/2, where “L” represents the larger diameter and “S” represents the smaller diameter. All animal experiments complied with the National Institutes of Health guide for the care and use of Laboratory animals (NIH Publications No. 8023, revised 1978).

### Statistical analysis

All data represent mean ± standard deviation. Statistical analysis was performed by Student's *t*-test using SPSS software. A *P* value < 0.05 was considered statistically significant.

## SUPPLEMENTARY MATERIALS FIGURES



## Abbreviations

TNF-α: Tumor necrosis factor-alpha
EMT: epithelial mesenchymal transition
CRC: colorectal cancer
KLT: Kanglaite
FBS: fetal bovine serum
MMP-9: matrix metalloproteinase-9
MTT: methyl thiazolyl tetrazolium
