# Supplementary material for: Kanglaite inhibits EMT caused by TNF-α via NF-κΒ inhibition in colorectal cancer cells
Source: Oncotarget. 2017 Dec 22;9(6):6771–9. doi: 10.18632/oncotarget.23645 (PMC5805513; doi:10.18632/oncotarget.23645)
Supplement: Supplementary file 1 [file oncotarget-09-6771-s001.pdf]

Kanglaite inhibits EMT caused by TNF-α via NF-κB inhibition in colorectal cancer cells

SUPPLEMENTARY MATERIALS

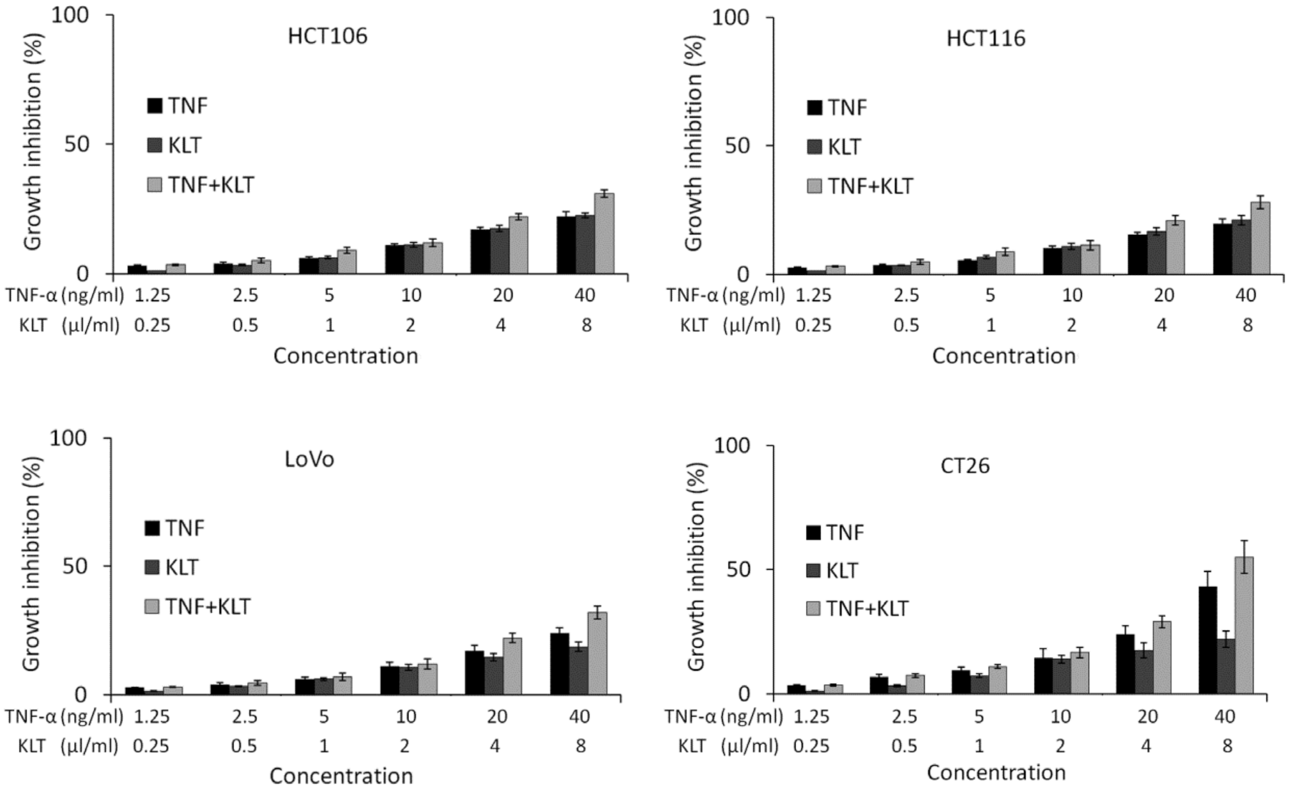

Supplementary Figure 1: MTT assay of the four CRC cell lines. The three experimental groups are single TNF-α or KLT treatment, and combined TNF-α and KLT treatment. Vertical axis represents growth inhibition rate compared with untreated cells; horizontal axis represents agent concentration. Experiments were performed in triplicate.

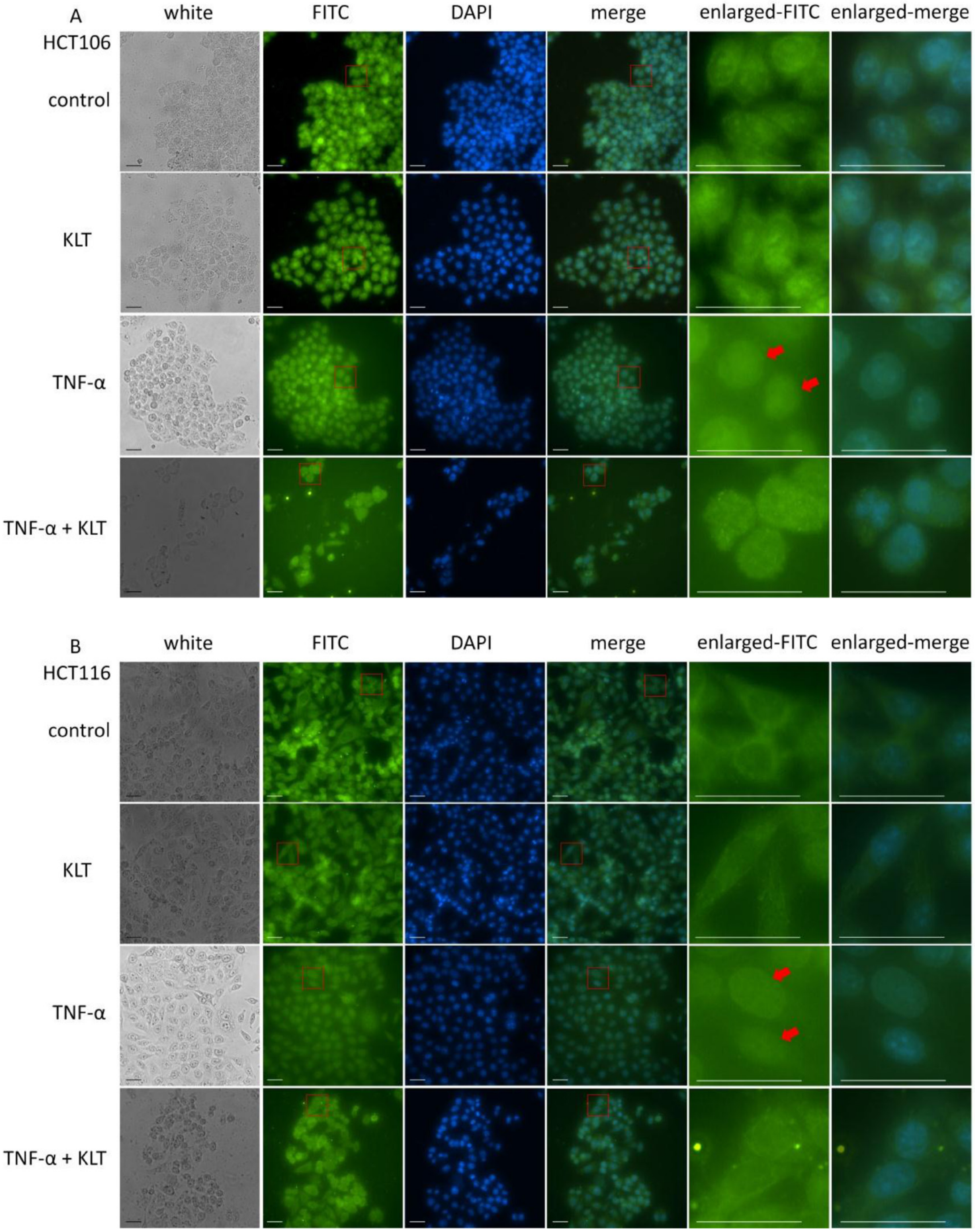
A
white
FITC
DAPI
merge
enlarged-FITC
enlarged-merge
HCT106
control
KLT
TNF-α
TNF-α + KLT
B
white
FITC
DAPI
merge
enlarged-FITC
enlarged-merge
HCT116
control
KLT
TNF-α
TNF-α + KLT

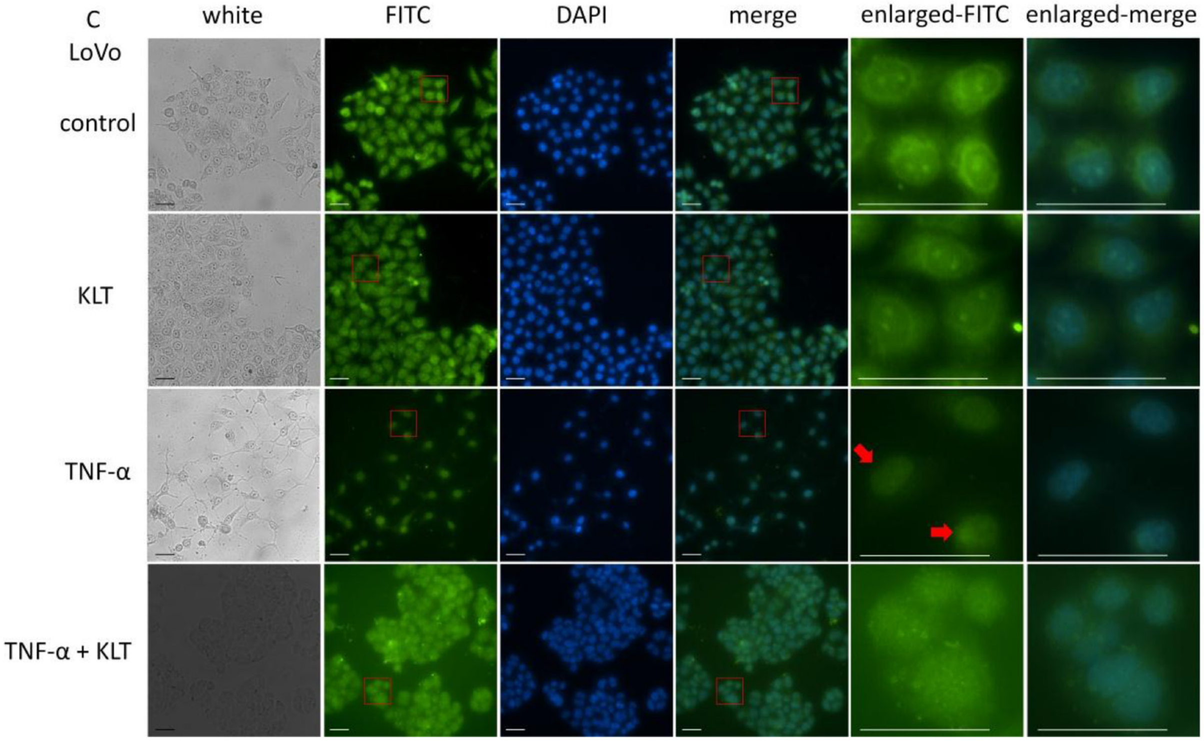

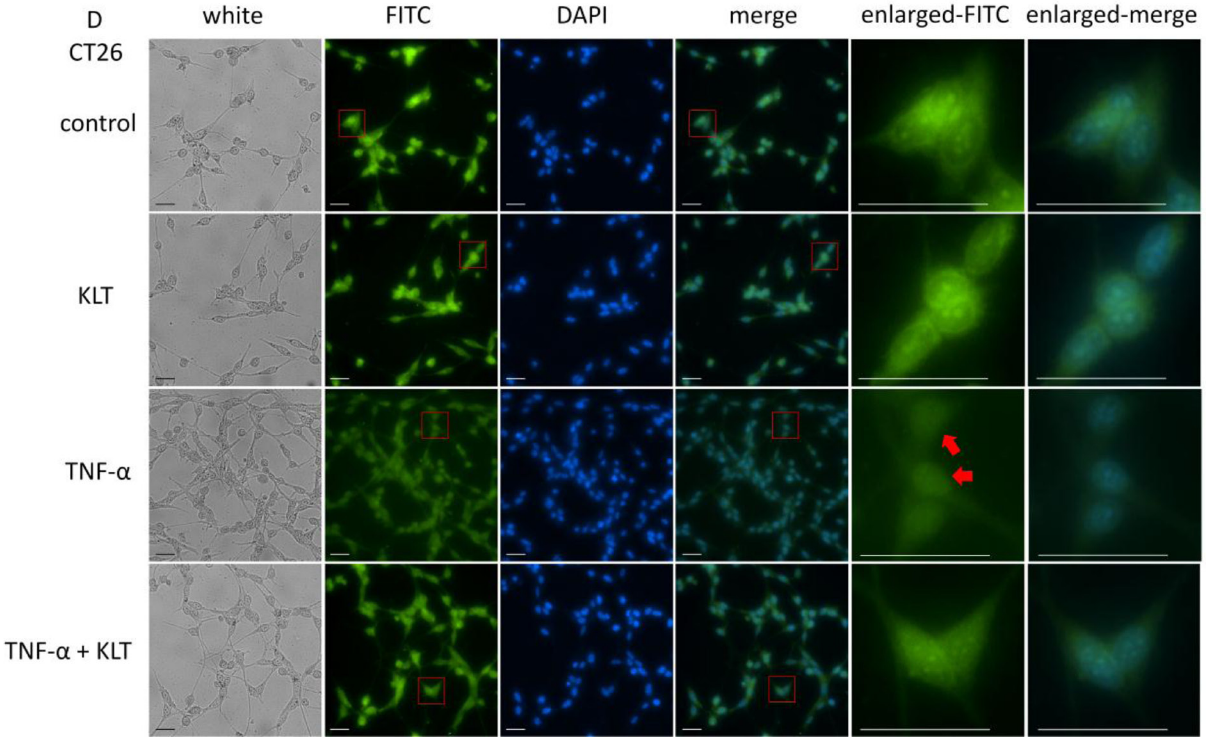

Supplementary Figure 2: Immunocytochemistry analysis of NF-κB p65. The four CRC cell lines is immunostained with NF-κB antibody followed by FITC-conjugated antibody. The drug treatment groups are listed in the left column. Activated NF-κB is highly expressed in the nuclei by TNF-α treatment, but the "TNF-α+KLT" group has not obvious NF-κB expression in the nuclei. The scale bars represent 20 μm. All images in red box are enlarged in order to determine whether NF-κB is translocated to nuclei. Red arrows indicate that NF-κB has high expression level in nuclei by comparison of FITC and DAPI stain. (**A**) HCT106; (**B**) HCT116; (**C**) LoVo; (**D**) CT26.

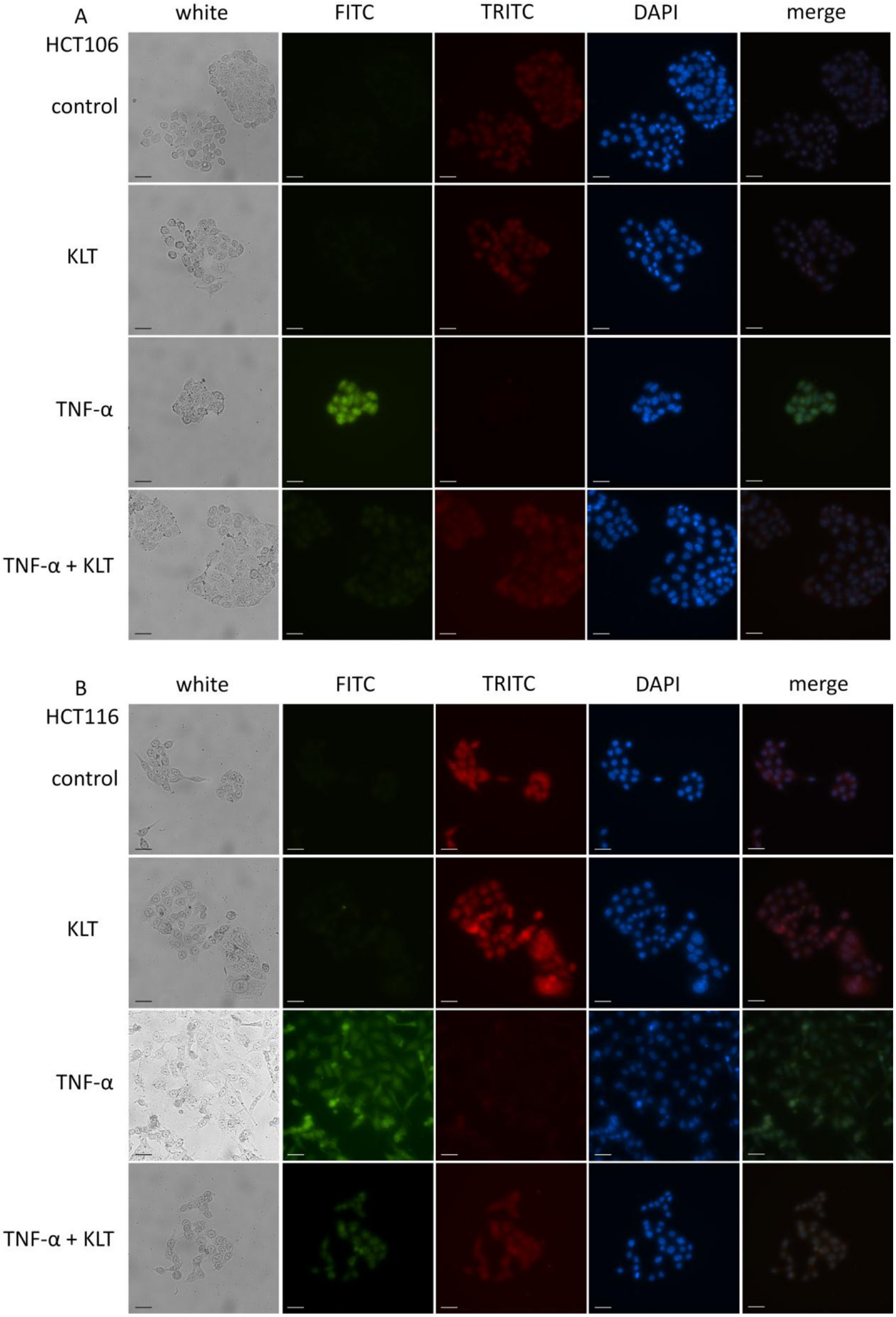
A
HCT106
white
FITC
TRITC
DAPI
merge
control
KLT
TNF-α
TNF-α + KLT
B
HCT116
white
FITC
TRITC
DAPI
merge
control
KLT
TNF-α
TNF-α + KLT

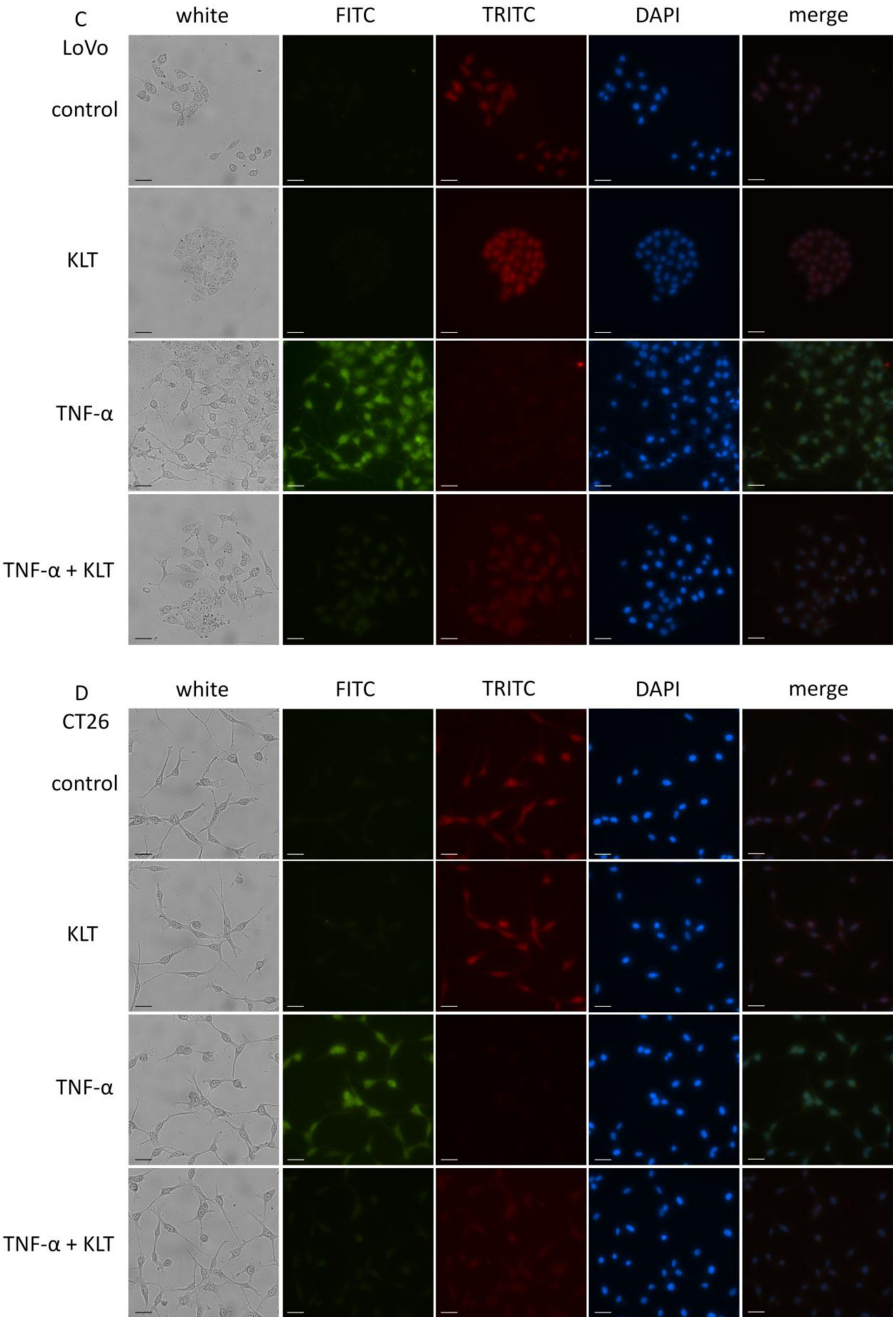

Supplementary Figure 3: Immunocytochemistry analysis of E-cadherin and vimentin in the four CRC cells. Double immunofluorescence staining of vimentin (FITC: green) and E-cadherin (TRITC: red) in the four CRC cells with dealing by different treatment listed in the left column of the image. The nuclei was stained with DAPI. The scale bars represent 20 μm. (**A**) HCT106; (**B**) HCT116; (**C**) LoVo; (**D**) CT26.

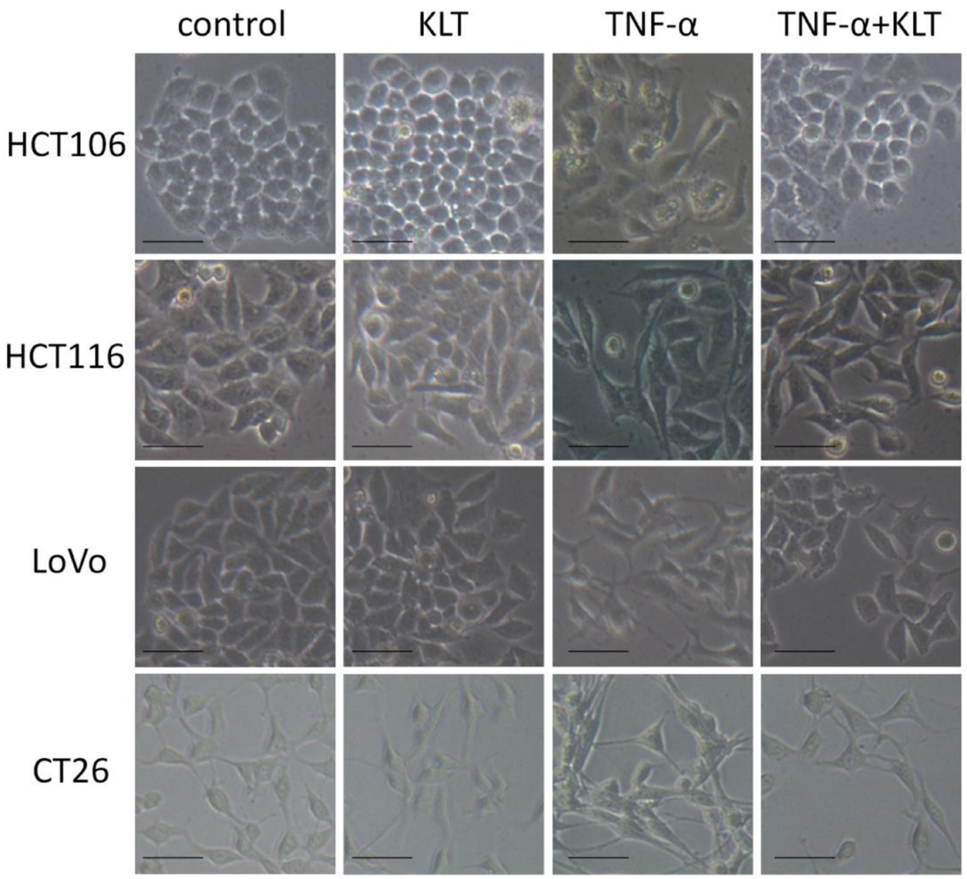

Supplementary Figure 4: Morphological change of the four CRC cells with dealing by different treatment listed in the left column of the image after 48 h. The scale bars represent 20 μm.

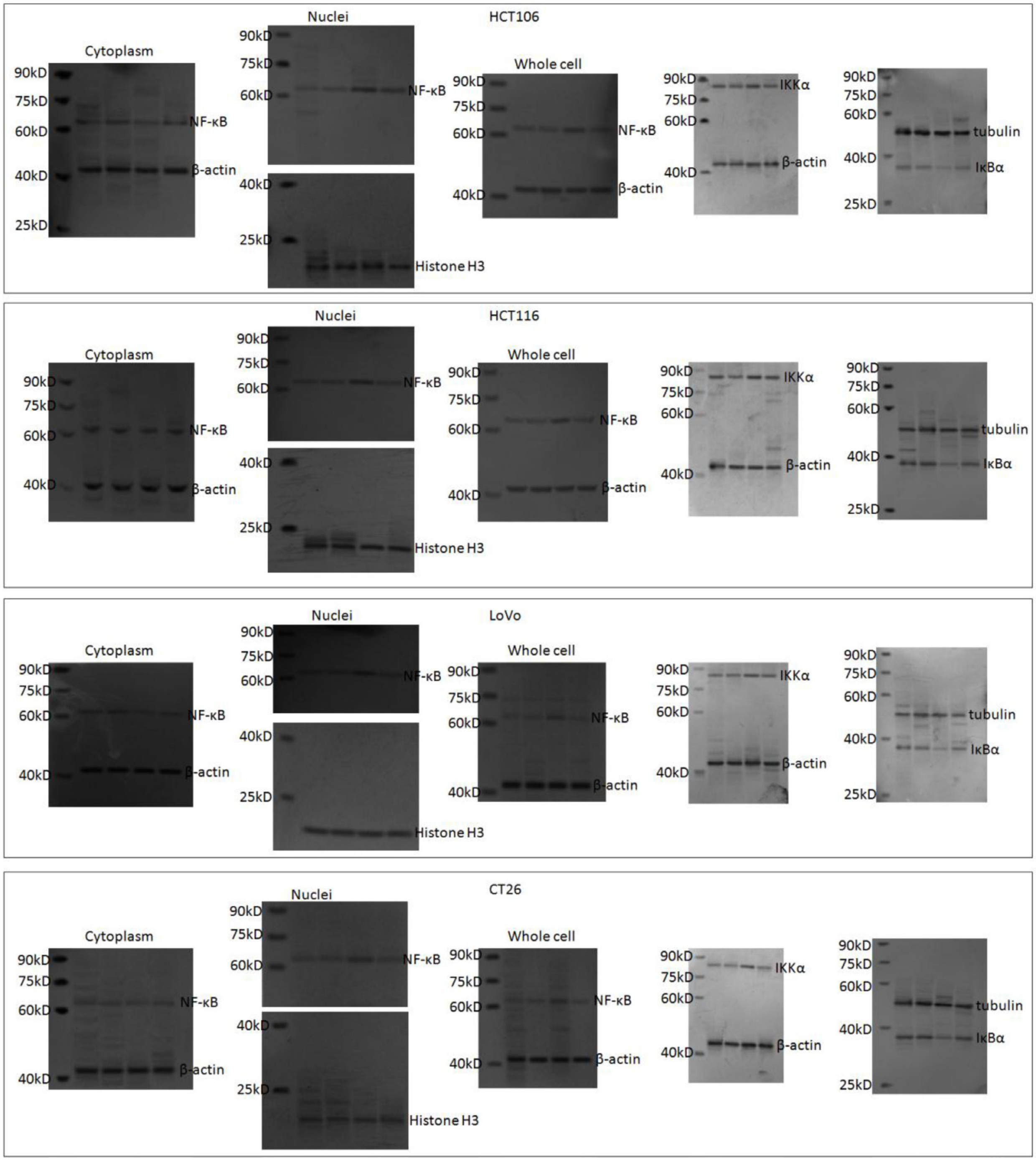

Supplementary Figure 5: Full length blots of Figure 1.

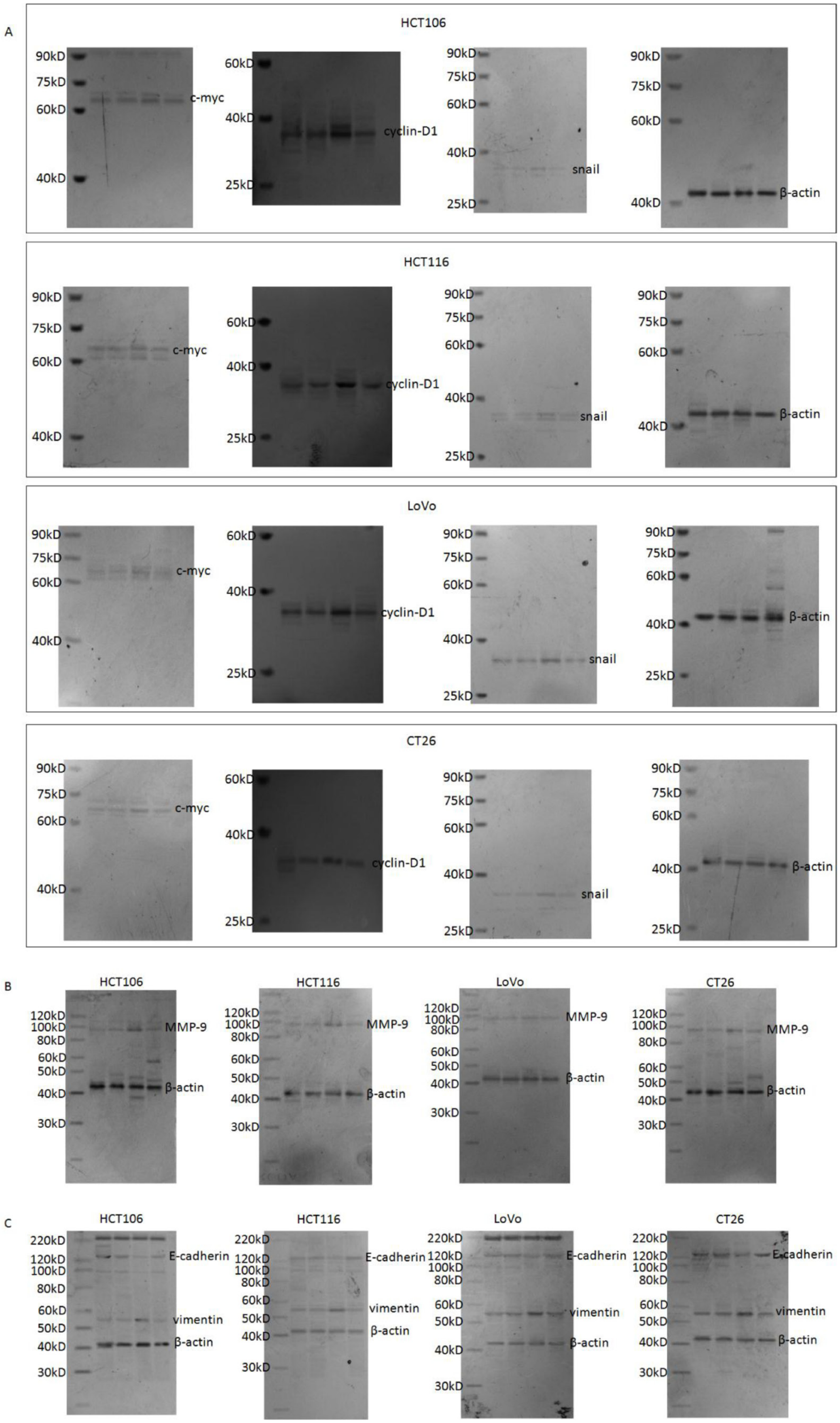

Supplementary Figure 6: Full length blots of Figure 2.
